# Proteasome Inhibitors and Their Potential Applicability in Osteosarcoma Treatment

**DOI:** 10.3390/cancers14194544

**Published:** 2022-09-20

**Authors:** Cassidy M. Van Stiphout, Anita K. Luu, Alicia M. Viloria-Petit

**Affiliations:** Department of Biomedical Sciences, Ontario Veterinary College, University of Guelph, Guelph, ON N1G 2W1, Canada

**Keywords:** osteosarcoma, proteasome inhibitors, bortezomib, carfilzomib, ixazomib, targeted therapy

## Abstract

**Simple Summary:**

Bone cancer has seen minimal benefits in therapeutic options in the past 30 years. Proteasome inhibitors present a new avenue of research for the treatment of bone cancer. Proteasome inhibitors impair the function of the proteasome, a structure within the cell that removes unwanted and misfolded proteins. Bone cancer cells heavily rely on the proteasome to properly function and survive. Impairing the proteasome function can have detrimental consequences and lead to cell death. This review provides a thorough summary of the in vitro, in vivo, and clinical research that has explored proteasome inhibitors for the treatment of bone cancer.

**Abstract:**

Osteosarcoma (OS) is the most common type of bone cancer, with ~30% of patients developing secondary/metastatic tumors. The molecular complexity of tumor metastasis and the lack of effective therapies for OS has cultivated interest in exploiting the proteasome as a molecular target for anti-cancer therapy. As our understanding towards the behavior of malignant cells expands, it is evident that cancerous cells display a greater reliance on the proteasome to maintain homeostasis and sustain efficient biological activities. This led to the development and approval of first- and second-generation proteasome inhibitors (PIs), which have improved outcomes for patients with multiple myeloma and mantle cell lymphoma. Researchers have since postulated the therapeutic potential of PIs for the treatment of OS. As such, this review aims to summarize the biological effects and latest findings from clinical trials investigating PI-based treatments for OS. Integrating PIs into current treatment regimens may better outcomes for patients diagnosed with OS.

## 1. Introduction

Osteosarcoma (OS) is the most common malignancy of bone in children and young adults [1,2,3,4]. It comprises approximately 30% of all bone sarcomas [5]. This spindle-shaped neoplasm consists of malignant mesenchymal cells that produce osteoid or immature bone [6,7]. While primary bone cancers are considered rare, OS is becoming increasingly more common [3,8,9,10]. The Canadian Cancer Society website includes the most recent incidence rate reported in Canada, stating that 240 Canadians were diagnosed with bone cancer in 2016 [https://cancer.ca/en/cancer-information/cancer-types/bone/statistics (accessed on 12 September 2021)]. The majority of cases are between 10–25 years of age [6], with OS onset being extremely rare before the age of 5 [1]. The age-adjusted incidence of OS is bimodal, with an initial peak in adolescence (18 years of age) and a secondary peak in patients over 60 years of age [6,11]. Arguably, males are reported to be affected more frequently (1.4:1 ratio of males to females, respectively) [12], and the incidence of OS in patients of African-American and Hispanic descent is slightly higher than in Caucasians [13].

In 80% of patients, tumor development arises in the metaphysis of long bones, specifically areas of rapid bone growth [5]. Approximately 40% of OS originates in the femur, 20% in the tibia, and 10% in the humerus [5]. Notably, patients over 25 exhibit an expansive range of primary tumor locations [14], as 20% of cases also present malignancies in the axial skeleton and soft tissue [5].

Most OS patients present local pain, with later swelling and limited joint movement [1,6,12,15]. In rare cases, more specifically in patients with osteolytic tumors [12], a pathological fracture can reveal emerging OS [6]. Evaluating a suspected OS patient begins with a full history, physical examination, and radiographs [12]. Patients are generally symptomatic for several months (average, 3–4 months, but often surpassing 6 months) before a conclusive diagnosis is made [1]. Reports indicate that a histological response to neoadjuvant therapy, a complete tumor resection, and metastases at diagnosis are vital prognostic factors [2,7].

OS is considered to be a systemic disease [6,11]. Tumor cells invade the circulatory system, and this can lead to fatal metastases [6]. Approximately 15–20% of patients present with radiographically detectable metastases at baseline [1,2]. These patients typically express the poorest prognoses, with reports of 5-year survival rates as low as 20% [5]. Besides synchronous metastases, about 40% of patients with localized OS develop secondary metastases [7]. Such outcomes have researchers postulating whether all patients have subclinical, microscopic metastases at initial diagnosis [1]. The most frequent site for metastatic presentation is the lung (>80% of cases) [2,16,17,18]; however, respiratory symptoms primarily develop only after extensive involvement [1]. Metastases can also arise in other bones and soft tissue [1]. Ward et al. reviewed high-grade OS patients with non-detectable metastasis at diagnosis that went on to develop metastases [19]. Survival rates displayed were 23% at 5 years and 0% at 4 years for pulmonary and bone metastases, respectively [19]. When OS undergoes extensive metastatic dissemination, particularly in recurrent cases, the disease can invade the central nervous system and regional lymph nodes [16,20]. Once terminal, long bone tumors tend to also metastasize to the heart, abdomen, and muscle [16].

In patients with relapsed and/or metastatic OS, metastasectomy has shown to provide a survival benefit [1,21]. Huang et al. also found that OS patients with a single metastatic nodule showed a better prognosis than those with multiple lung nodules [21]. Furthermore, patients who developed metastases after completing the chemotherapy protocol had a better prognosis compared to those who had metastases identified at the initial presentation [21].

## 2. Breaking a 30-Year Plateau in OS Treatment

In the past thirty years, many trials have sought to establish a gold-standard therapy for primary, high-grade, and intramedullary (conventional) OS with non-detected metastasis at diagnosis, which represents approximately 80% of all osteosarcomas [11,22]. Current regimens encompass primary (preoperative; neoadjuvant) induction chemotherapy, followed by definite surgery and then postoperative (adjuvant) chemotherapy [12]. Chemotherapeutic agents commonly used to treat OS include methotrexate, doxorubicin (adriamycin), cisplatin (platinol), ifosfamide, and etoposide [6,7,23].

This multi-agent treatment has dramatically improved outcomes for patients with localized OS, with long-term survival rates improving from less than 20% to >60% [2,5]. However, limited therapeutic progress has been made since that time. Clearly, a novel therapeutic strategy is needed to improve care and overall patient survival. As our understanding towards the behavior of malignant cells expands, it is evident that cancerous cells, including OS cells, display a greater reliance on the proteasome to maintain homeostasis and sustain efficient biological activities [24]. Identifying the proteasome’s role in cell survival, proliferation, and response to standard treatment of OS cells, specifically metastatic or metastasis-prone populations, will aid in the development of effective proteosome inhibitors (PIs). This review aims to summarize the biological effects and latest findings from clinical trials investigating PI-based treatments for OS and provides justification for integrating PIs into current treatment regimens for OS patients.

## 3. Protein Homeostasis Requires the Ubiquitin Proteasome System

Protein homeostasis (proteostasis) plays a vital role in cell survival. The timely degradation of cyclin-dependent kinase (CDK) activators or inhibitors is necessary for the cell to advance through all stages of the cell cycle, from DNA replication to mitosis [25,26]. Active proteosomes are also essential for cell regulation and the degradation of misfolded or mutated proteins [25,26,27,28,29].

The ubiquitin–proteasome System (UPS) regulates cellular functions by removing damaged or misfolded proteins from the cell [26]. Ubiquitin is a short protein consisting of 76 amino acids [30,31,32]. The process of ubiquitylation occurs through the continuing participation of three class proteins: the ubiquitin-activating enzyme (E1), ubiquitin-conjugating enzyme (E2), and ubiquitin protein ligase (E3) [30]. Initially, this process is activated when E1 links to the C-terminal glycine residue of ubiquitin through the formation of a high-energy thiol ester with an internal E1 cysteine residue [33,34]. Following this, E2 transfers the activated ubiquitin from the E1–ubiquitin complex to a cysteine residue situated in the E2 enzyme [33,34]. Finally, E2 interacts with the ligase E3, which catalyzes the formation of a peptide bone between a carboxyl group at the C-terminus of the ubiquitin and an amine group of the substrate [33,34]. Notably, E2 can interact with several E3s in a substrate-specific manner [35]. In most cases, this process is repeated until a polyubiquitin chain emerges, targeting the protein for degradation in the proteasome [30].

**Figure 1:**
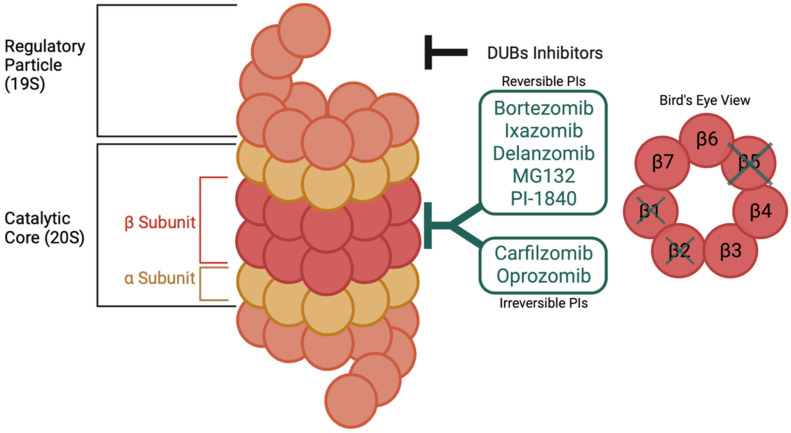
Overview of proteasome structure and target sites of inhibitors. The proteasome’s multiprotein complex is composed of a catalytic core and regulatory particles. The majority of clinically used compounds preferentially target the β5 site of the 20S catalytic core (e.g., Reversible PIs, Irreversible PIs). However, compounds directed towards the 19S regulatory subunit of the proteasome, generally targeting deubiquitinases (DUBs), are currently under investigation. Since they bind to an alternative site on the proteasome, they would be particularly useful in overcoming resistance to compound targeting the 20S core.

The 26S proteasome is a multiprotein complex that mediates protein degradation (Figure 1). It is composed of two components: the catalytic core, also called the 20S, and one or two 19S regulatory subunits, also called the 19S regulatory particle (RP) or PA700, on either end of the 20S core [26,36,37,38]. The 19S subunit binds to the polyubiquitin chain, which cleaves it from the target protein [26]. The ATP-dependent interaction between the 19S subunit and the catalytic core allows the pores of the 20S proteasome to open [37]. This provides an access portal for substrates to the catalytic core [37]. The protein then passes through the 20S core where it is degraded to small oligopeptides of 3–25 amino acids in length [27].

The 19S subunit usually borders the 20S core, though the core can act alone to cause ubiquitin-independent protein degradation [26,27,39]. The 20S particle is a cylindrical structure composed of four heptameric rings [26,27,36]. The two outer rings (α rings) flank the two inner rings (β rings) [26,36]. Each β rings contains three active sites for protein degradation: β5 (chymotrypsin-like; CT-L), β2 (trypsin-like; T-L), and β1 (caspase-like, C-L) [26,36]. The β5 site is the primary target of PIs; however, at higher concentrations of PI drugs, β2 and β1 sites are inhibited as well [26].

## 4. Proteasome Inhibition in Cancer

Empirical evidence demonstrates enhanced tumor cell sensitivity to proteasome inhibition compared to normal cells [40]. Being highly proliferative, tumor cells have an increased requirement for protein synthesis, which enhances their vulnerability to proteasome inhibition [40]. Such inhibition prevents proteasome substrates from being degraded, which subsequently leads to cell death. Many of these proteasome substrates include signaling molecules, tumor suppressors, cell cycle regulators, transcription factors, inhibitory molecules (whose degradation activates other proteins), and anti-apoptotic proteins (e.g., Bcl-2) [27]. Significant PI-induced apoptosis has been reported in numerous tumor cell types relative to their corresponding non-cancerous counterparts, including in human chronic lymphocytic leukemia, oral squamous cell carcinoma, human multiple myeloma, and human PC-3 prostate cells [25,40].

**Figure 2:**
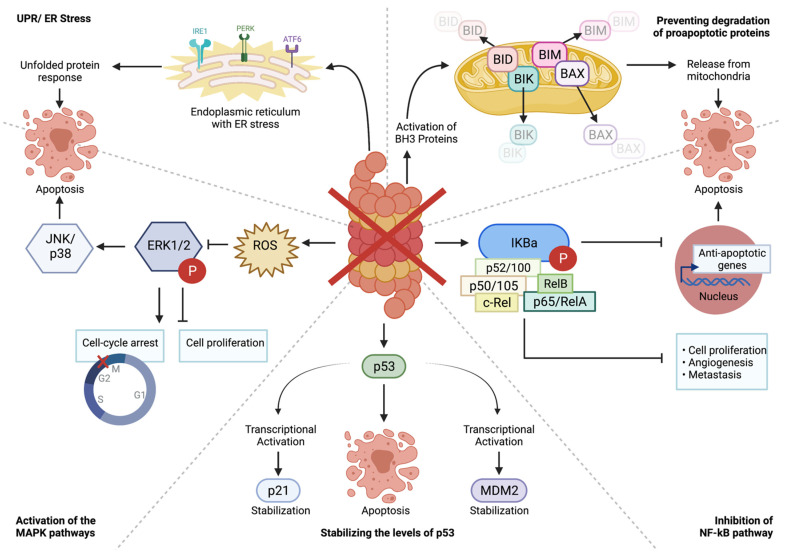
Cellular mechanisms by which proteasome inhibition triggers apoptosis.

A description of the pathways that are impacted by proteasome inhibition are explained below and summarized in Figure 2.

### 4.1. PI’s Mechanisms of Action 

#### 4.1.1. Inhibition of NF-κB Pathway

PI-based therapy began to cultivate interest after they displayed inhibitory effects on the nuclear factor-kappa B (NF-κB) pathway, a pro-survival pathway for various cell types, including those of osteoid lineages [26,41]. The NF-κB pathway assists in controlling inflammation, oncogenic transformation, tumor progression, and the acquisition of resistance to standardized chemotherapeutic agents [42]. To activate the pathway, stimuli, such as TNF-α and oxidants [41], are received by IκBα, a NF-κB protein inhibitor with proteasome-dependent degradation [26,27,43]. Following this, IκBα is phosphorylated and subsequently degraded, which allows NF-κB proteins to become active in the cytoplasm and translocate to the nucleus (Figure 2) [26,27,41,42]. These proteins, namely p50/p105, p52/p100, p65/RelA, c-Rel, and RelB, modulate transcription of targeted genes to prevent pro-apoptotic machinery from being activated [26,41,43]. Genes targeted may include the anti-apoptotic Bcl-Lx, cFLIP, cIAP1/2, and Bcl-2, and the antioxidants superoxide dismutase and the ferritin heavy chain [42]. However, when the proteasome is inhibited, IκBα remains intact and bound to NF-κB proteins, thus preventing the activation of the NF-κB pathway [26,43].

Recently, OS cell lines were reported to present a mechanism of cisplatin resistance by enhancing the protein expression of NF-κB molecules [44]. The binding of RelA to Wee1, a kinase inhibitor of CDK activity, allows RelA to translocate into the nucleus [44]. Downstream signaling of RelA activity then increases Bcl-2 expression, while also suppressing the apoptotic-related genes poly-ADP ribose polymerase (PARP) and caspase-3 [44]. PI treatment of OS tumors of this molecular signature can not only interfere with the tightly controlled protein homeostasis, but also restore cisplatin drug sensitivity. Such interference results in heightened levels of specific signaling molecules, such as the NF-κB inhibitor IκB and CDK inhibitors p21 and p27, and causes the build-up of misfolded proteins that can trigger apoptosis [45]. This is supported by a model by Zhang et al., where the deactivation of the NF-κB pathway causes the accumulation of p21, thereby leading to cell cycle arrest at the S phase in human OS cells [46].

#### 4.1.2. Activation of the MAPK Pathways

Other mechanisms of cellular toxicity have been proposed for PIs. The mitogen-activated protein kinase (MAPK) pathways encompass the extracellular signal-regulating kinase (ERK1/2), the Jun-N-terminal kinase (JNK), and the p38 MAPK pathways which modulate cell proliferation, stress responses, and survival, respectively [47,48]. In general, JNK and p38 MAPK activation is associated with apoptosis induction, whereas ERK activation is associated with cytoprotection [47]. Studies suggest that perturbations in MAPK pathways may be involved in regulating PI-mediated lethality [46]. Proteasome inhibition with MG132 resulted in reactive oxygen species (ROS) production [49]. This results in ERK1/2 inactivation, causing JNK/p38 activation followed by apoptosis in human OS cells (Figure 2) [26]. In addition to inducing cell cycle arrest at the G_2_/M phases, Lou et al. showed concentration-dependent inhibition of ERK phosphorylation by bortezomib in OS cells [47]. This suggests that PIs may inhibit cell proliferation via inhibition of ERK phosphorylation, and that growth inhibition is mediated, at least in part, by inhibiting MAPK pathways in OS cells.

#### 4.1.3. Stabilizing the Levels of p53

The p53 gene is a known tumor suppressor, functioning to stop growth or to activate cell death under diverse circumstances. It is well documented that mutations in p53 contribute to OS development [50]. Initial research on p53 status in OS noted that mutations were only detected in 20% of OSs. More recently, Synoradzki et al. suggested that this frequency is much higher—from 47–90% [51]. Studies comparing primary OS tumors to their paired metastases using whole exome sequencing and phylogenetic analysis found ubiquitous loss of heterozygosity in chromosome region 17p (harboring TP53) in primary and secondary tumors, suggesting that loss of p53 is an early event during OS progression [52]. This may explain why studies examining the potential of p53 as a prognostic marker in OS patients have failed to reach consensus on a prognostic role [50].

The expression of p53 is notably stimulated by PI-based treatments. Studies in various cell lines have found that PI treatments result in the stabilization and rapid accumulation of p53 [26,53], leading to the transactivation of p53 target genes encoding p21 and MDM2 [48,54]. Proteasome inhibition can prevent MDM2-mediated p53 ubiquitination, which subsequently activates the JNK pathway and causes cell death (Figure 2) [26,30]. Lopes et al. found that PI-induced apoptosis was blocked by the expression of dominant-negative p53, yet overexpression of wild-type p53 was sufficient to induce apoptosis [53]. Similarly, Lauricella et al. found that wild-type p53 expression potentiates the apoptotic effect induced by MG132 in OS cells [49]. These findings suggest that modulation of p53 turnover is a key event in PI-induced apoptosis.

Although PI-mediated cell death has shown to depend on p53 expression in some cell lines, inconsistencies regarding the role of p53 do exist [26,27]. Additional studies have recorded that PIs induce p53-independent expression of the pro-apoptotic BH3-only member of the Bcl-2 family, NOXA, but not the PUMA protein [26]. It was demonstrated that PIs inhibit the growth of cancer cell lines independently of p53 mutation status [55]. A mutation of this gene appears to endow pro-tumorigenic effects on cancer cells, leading to chemotherapeutic resistance and promoting OS metastases [26]. Since PI-based therapy is effective regardless of p53 status, it provides an alternative to chemoresistant tumors.

#### 4.1.4. Preventing the Degradation of Pro-Apoptotic Proteins

PIs can indirectly trigger apoptosis by preventing the degradation of pro-apoptotic proteins. In correspondence to the stabilization of p53, pro-apoptotic BH3-only proteins have been shown to be transcriptionally upregulated in response to cellular stresses such as DNA damage induced by hypoxia, growth factor deprivation, or mitogenic stimulation [30]. However, under normal cell conditions, these proteins, including BIM, BID, and BIK, are regulated through rapid ubiquitination and proteasomal degradation [26]. Upon proteasomal inhibition, these proteins accumulate, resulting in caspase-9, -8, and -3 activation, and subsequent cell death (Figure 2) [26]. Liu et al. noted that treatment of OS cells with ixasomib demonstrated significant dose-dependent induction of BID activity, as indicated by an increase in cleaved form t-BID, thus triggering caspase-dependent apoptosis [24].

Interestingly, NOXA has shown to interact with pro-apoptotic effectors BAX and BAK of the Bcl-2 family in the presence of PIs [30]. The direct binding of NOXA facilitates the oligomerization of BAX and BAK, causing the release of mitochondrial intermembrane space proteins such as cytochrome c into the cytosol [26,30]. Cytochrome c release enables the formation of the apoptosome followed by cleavage of initiator and effector caspases, executing apoptotic cell death [30,56]. More recently, expression levels of BAX, cleaved caspases, and cleaved PARP were increased dose-dependently in PI-1840-treated OS cells [57]. Moreover, the level of cytochrome c in the mitochondria decreased, which confirmed the presence of apoptosis in the OS cells at the mitochondrial level [56]. These findings indicate that PIs induce apoptosis in OS cells by activating both extrinsic and intrinsic pathways [56]. Of note, PI-1840 is an exclusive non-covalent inhibitor, which confers chemical stability and reduced reactivity compared to all other covalent-binding PIs [58,59].

#### 4.1.5. Modulation of TRAIL

PI-stimulated apoptosis has also been associated with the upregulation of tumor necrosis factor (TNF)-related apoptosis-inducing ligand (TRAIL) and its death receptors (DR), DR4 and DR5 [26,30]. TRAIL is a cytokine belonging to the TNF family of ligands [30], which mediates apoptotic effects by binding to the death receptors [60]. Proteosome inhibition upregulates TRAIL binding to DR5. As such, the cooperation between PIs and TRAIL increases apoptotic activity (Figure 2) [57]. Li et al. observed that the combination of MG132 treatment and TRAIL elevated levels of DR5, caspase-3/-8, induced apoptosis, and suppressed the invasiveness of OS cells [61].

#### 4.1.6. Proteotoxic Crisis, Endoplasmic Reticulum (ER) Stress, and the Unfolded Protein Response (UPR)

The chaotic genome and overactivity of various signaling pathways (i.e., mTOR) encourage a heightened rate of protein synthesis in cancer cells [62,63]. In the case of OS, this increased protein production has been proven to be useful and provide cells with a competitive metastatic advantage during periods of stress. Morrow et al. found that highly metastatic OS cells translate more proteins as they arrive, invade, and colonize the lung microenvironment [64]. However, this excessive protein synthesis is not always advantageous and can lead to a proteotoxic crisis within cancer cells. Following protein synthesis, nascent proteins travel to the endoplasmic reticulum (ER) for proper protein folding and eventually leave the ER for further modifications prior to dispatching to the final destination. In the instance of cancer, protein production is excessive and protein products are often mutated due to point mutations in protein-coding regions [63]. These mutated proteins lead to challenges in protein folding, accumulate in the ER lumen, and eventually overload the capacity of the ER, leading to “ER stress” [65]. To maintain cell homeostasis, cells initiate an unfolded protein response (UPR) which involves three signaling pathways: inositol-requiring enzyme 1 (IRE1), double-stranded RNA-activated protein kinase (PKR)-like ER kinase (PERK), and activating transcription factor 6 (ATF6) [66]. These work in parallel to ameliorate ER stress by transcribing UPR target genes (i.e., heat shock proteins to aid in protein folding in ER), inhibiting mRNA translation through phosphorylation of eIF2α, and increasing the activity of transcription factors ATF4 and spliced X-box binding protein 1 (XBP1). ATF4 drives the transcription of C/EBP homologous protein (CHOP) and growth arrest and DNA damage-inducible 34 (GADD34) [62]. The transcription factor CHOP regulates the expression of genes involved in apoptosis, while GADD34 recruits protein phosphatase 1 (PP1) to dephosphorylate eIF2α to eventually restore protein synthesis [67]. Lastly, XBP1 regulates the expression of ER chaperones, lipid synthesis enzymes, and ER-associated degradation (ERAD) proteins. Altogether, these mechanisms aim to increase the protein-folding capacity in the ER and decrease the stress within the ER by inhibiting protein translation and degrading misfolded proteins through coupling with the UPS [68]. UPR is meant to be adaptive and restore cell homeostasis. However, if this stress is prolonged or is insufficient in alleviating the stress, UPR can be fatal, and cells undergo apoptosis (Figure 2). Obeng et al. found that bortezomib induces a terminal UPR in multiple myeloma cells, emphasizing the importance of the UPS system in proteostasis [69].

## 5. PIs Used in Cancer Treatment and Evidence for Their Use in OS

The purpose of developing PIs was initially to provide a potential benefit in attenuating cancer-related cachexia [41]. However, after many preclinical studies, it was evident that small-molecule PIs could induce apoptosis in cultured cell lines and cancer models [40,70,71]. Thus, their utility as a chemotherapeutic agent was postulated [40,70,71]. This rationale led to the development of bortezomib [40,71], a first-generation proteasome inhibitor, and then, later, second-generation agents, including carfilzomib and ixazomib, which were developed to improve the benefits observed with bortezomib [41,72,73]. These three agents inhibit the 20S catalytic core of the proteasome and are currently approved for the treatment of multiple myeloma (MM), while bortezomib is also used in the treatment of mantle cell lymphoma (MCL) [40,72,74]. Since then, other proteasome inhibitors, such as oprozomib and delanzomib, have been discovered [45].

To date, research evaluating PIs in OS are predominately preclinical and have employed both human and canine models [45]. Being in close quarters with humans, canines are exposed to similar environmental factors [75,76]. They offer a natural way to study the disease and are evolutionarily closer to humans in comparison to rodent models [75,76]. Similar to humans, OS in canines is also considered uncommon; however, it occurs much more frequently in canines compared to humans [77]. While the incidence rate in humans is estimated to be 1.02/100,000, the incidence in canines is 13.9/100,000 [78]. OS is particularly common in larger breeds and arises primarily in the appendicular skeleton, often within the metaphyseal region of the long bones [45]. Metastases tend to occur faster, but also develop in the lungs, which is universally fatal [45,77]. The genetic and molecular biology of OS exhibits a high degree of overlap in both species, making canines a great naturally occurring translational model in the study of OS [78,79,80]. Research conducted in either species has the potential to expand our understanding on OS biology and provide insight on promising therapies in the other. In the section below, we summarize in vitro, preclinical (usually xenograft models in mice), and clinical research that has explored PIs in both human and canine OS.

### 5.1. First-Generation PI: Bortezomib

Bortezomib was the first proteasome inhibitor approved for clinical use (Table 1) [73]. Bortezomib is a modified dipeptide boronic acid that binds selectively and reversibly to the 26S proteasome [81]. It forms a coordinate covalent bond of high affinity to the β5 (CT-L) [73]. However, binding to β1 (C-L) and β2 (T-L) subunits with lower affinity has been observed as well [73]. The direct binding of bortezomib inhibits the 26S, which triggers the apoptotic signaling cascade [81].

#### 5.1.1. In Vitro

Preclinical development reveals that canine and human OS cell lines are extremely sensitive to bortezomib in vitro [45]. Patatsos et al. used a panel of four canine OS cell lines to evaluate the sensitivity to physiologically achievable concentrations of chemotherapeutic drugs currently used to treat OS (doxorubicin and carboplatin) in combination with bortezomib [45]. Bortezomib potently induced caspase-dependent apoptosis at a considerably lower concentration than that found in the bones and lungs of treated rodents [45]. Co-treatment with bortezomib, plus either doxorubicin or carboplatin, displayed higher toxicity to canine OS cells than each agent alone [45]. Bortezomib combined with carboplatin appears to be synergistic at high doses, while bortezomib combined with doxorubicin was only weakly synergistic, tending towards antagonistic at high doses [45]. These findings suggest that the addition of bortezomib to existing regimens may be beneficial, albeit only at certain concentrations.

Subsequently, Patatsos et al. demonstrated that human OS cells were as sensitive to bortezomib as canine cells [45]. Additional work by Lou et al. also found that bortezomib suppressed tumor growth, autophagy, and apoptosis in a human OS cell line [47]. Noteworthy, a review by the European Medicines Agency has acknowledged findings suggestive of bortezomib tolerance in canines being better than humans. The maximal tolerated dose in humans is 1.3 mg/m^2^ but 3.6 mg/m^2^ in dogs [45]. Thus, canines present a valuable model to study underlying mechanisms, but it is important to consider that they do not fully recapitulate all aspects of OS. In vitro findings could be impacted due to species variability in what may be physiologically achievable.

#### 5.1.2. In Vivo

The induction of apoptosis and suppressed growth of human OS cells was also observed in vivo by Shapovalov et al. [82]. OS 143B cells expressing luciferase (143B-luc) (5 × 10^4^) were injected orthotopically into the medullar cavity of right tibiae of 5-week-old nude mice [82]. Eight days after tumor cell injection, control mice received PBS, while treated mice received 1 mg/kg of bortezomib intraperitoneally (i.p.) every 3 days for 3 weeks [82]. A significant 70% reduction in tumor size was observed in the bortezomib group at day 28 of treatment [82]. Immunohistochemical analyses in OS xenografts revealed that bortezomib inhibited cell proliferation and induced apoptosis, which correlated with increased immunoreactivity for BAX [82].

In a subsequent study, the effect of bortezomib in combination with doxorubicin was evaluated in a different human OS xenograft mouse model [84]. Nude mice were subcutaneously inoculated with 5 × 10^6^ OS KHOS/NP cells and then randomized to receive one of the following treatments i.p. twice a week for 17 days: vehicle control (1% dimethyl sulfoxide, 7% cremophor/ethanol (3:1), and 92% phosphate-buffered saline), doxorubicin (0.5 mg/kg, IP), bortezomib (0.2 mg/kg, IP), or a combination of doxorubicin and bortezomib (0.5 mg/kg doxorubicin plus 0.2 mg/kg bortezomib) [84]. The combination therapy exhibited a potent synergistic effect, with tumor volume being significantly blunted compared to any agent used alone [84]. Bortezomib combined with doxorubicin induced activation of the ROS and the *p*-eIF2α/ATF4/CHOP signaling axis in the UPR pathway [84]. Thus, the addition of bortezomib to doxorubicin might improve OS treatment.

#### 5.1.3. Clinical

Although bortezomib revolutionized the treatment of human MM and MCL, it has not been used to treat canine patients [45]. Furthermore, only a handful of studies have documented the ability of bortezomib as a sole agent to kill human OS cells [45]. In a phase II study of recurrent metastatic patients (N = 21), bortezomib was administered at 1.5 mg/m^2^ by intravenous push twice weekly for 2 weeks, followed by 1 week of rest [83]. The dose was escalated to 1.7 mg/m^2^ if patients tolerated the first cycle [83]. Only one patient with leiomyosarcoma confirmed a partial response [83]. One OS patient was included but their response was not specified [83]. Two pediatric OS patients received bortezomib in a different phase I dose escalation study without experiencing objective responses [102]. Both studies concluded that bortezomib had minimal activity in these contexts as a single agent. Bortezomib is currently being investigated alone (NCT 00027716; trial completed but results unavailable) and in combination with the chemotherapeutic agents gemcitabine and doxorubicin (NCT 00479128) in patients with advanced or metastatic urothelial cancer or other solid tumors [85,103].

### 5.2. Second-Generation PI: Carfilzomib

Carfilzomib is a second-generation PI that received fast-track FDA approval in 2012 (Table 1) [87]. This drug displayed high efficacy and safety results for relapsed and/or refractory MM patients [87], even in those that received prior bortezomib therapy [104]. The development of carfilzomib was based on the proteasome being characterized as a major target of the natural product epoxomicin [87,104]. The synthesis of a biotinylated chemical probe led to the discovery that the epoxyketone group of epoxomicin covalently binds to the proteasome, selectively choosing it over other types of proteases [87]. This prompted the modification of YU-101, a leading epoxomicin analog with potent anticancer activities, to yield carfilzomib [87].

#### 5.2.1. In Vitro

The sensitivity of canine and human OS cell lines to carfilzomib has also been tested in vitro [45,88]. Carfilzomib manifested similar effects in canine OS cells to that seen in bortezomib [45]. However, unlike bortezomib, human OS cells were slightly less sensitive to carfilzomib compared to canine OS cells [45]. More recently, Somarelli et al. also found carfilzomib and bortezomib to demonstrate high efficacy across nine OS cell lines, which were of both canine and human origin [88]. Both inhibitors caused an average rate of cell death >95% in all nine cell lines [88].

Recurrent metastatic solid tumors are most recognized for causing high mortality rates, particularly in pediatric patients [91]. In efforts to improve outcomes, Thakur et al. tested the effectiveness of carfilzomib in killing tumor cells that have acquired treatment resistance and metastatic properties [91]. A panel of pediatric solid tumor cell lines, including OS cells, were treated with carfilzomib, which elicited cytotoxicity against all cell lines [91]. When carfilzomib was combined with chemotherapeutic agents, the inhibitor synergistically enhanced the extent of cell death [91]. To our understanding, this study provides initial in vitro data on the potential of carfilzomib to treat pediatric solid tumors.

Studies have also explored carfilzomib in combination with other molecular-targeted therapies. Carfilzomib-induced cell apoptosis was synergistically enhanced when combined with MAPK inhibitors U0126, SP00125, or SB203580 [94]. It was found that the combinational inhibition of ERK1/2 or JNK MAPK pathways significantly decreased the expression of anti-apoptotic Bcl-2 proteins, suggesting a new promising strategy to test clinically [94]. An in vitro analysis of OS cell lines for sensitivity to an array of approved cancer therapies has also revealed histone deacetylase (HDAC) inhibitors as being highly effective at triggering OS cell death [8]. Carfilzomib was tested in combination with HDAC inhibitors romidepsin and panobinostat [8]. Interestingly, for panobinostat and carfilzomib, a synergistic effect was achieved when the drugs were administered together [8]. However, for carfilzomib and romidepsin, the results suggest that the best synergy is achieved when applying the HDAC inhibitor either prior or concurrently to proteasome inhibition [8].

#### 5.2.2. In Vivo

The efficacy of combinational therapy tested in vitro led McGuire et al. to further explore panobinostat with carfilzomib in vivo [89]. Each reagent was examined alone and in combination on the growth and metastasis of OS [89]. Luciferase-expressing OS cell lines K7M2 and SAOS2-LM7 were injected into BalB/c or NSG mice, respectively. To examine OS growth, 1 × 10^5^ cells were injected intratibially [89]. To observe metastases, 1 × 10^6^ K7M2 cells were injected intravenously by tail vein injection [89]. Carfilzomib alone, given at 2 mg/kg by tail vein injection on 2 consecutive days, followed by 5 treatment-free days, had no effect on primary OS growth [89], and when given in combination with HDAC inhibitor panobinostat (0.2 mg/kg carfilzomib and 1 mg/kg panobinostat), it attenuated the beneficial effects of panobinostat [89]. Furthermore, carfilzomib alone had no beneficial effect on spontaneous lung metastasis but did not hinder the panobinostat efficacy when used in combination [89]. These data highlight the need for in vivo testing of potentially synergistic therapies identified in vitro but do support the use of HDAC inhibitors for the treatment of primary and metastatic OS [89].

#### 5.2.3. Clinical

Carfilzomib treatment has been associated with hepatic impairment of varying degrees, from mild to severe [90,105]. In efforts to understand the pharmacokinetics (PK) and safety of this PI drug, Brown et al. examined carfilzomib in patients with relapsed or progressive advanced malignancies [90]. Patients with normal or impaired hepatic function (mild, moderate, or severe) received carfilzomib infusion in 28-day cycles [90]. Carfilzomib treatment had a higher predicted probability of increasing hepatic impairment in patients with mild and moderate hepatic impairment [90]. However, these increases were deemed unlikely to be clinically significant, due to the intrinsic PK variability and inconsistent relationship in carfilzomib exposure response [90].

Recently, phase I/II clinical trials (NCT 00531284, NCT 02257476, NCT 00884312) have further evaluated the safety, tolerability, PK, and anti-tumor activity of carfilzomib in patients with advanced or relapsed solid tumors [92,93,106]. For instance, a phase I study (NCT 02257476) aimed to find the safety of weekly administration, testing carfilzomib at an initial dose of 20 mg/m^2^ on days 1, 8, and 15 of a 21-day cycle compared to the typical dosing schedule of days 1, 2, 8, 9, 15, and 16 of a 28-day cycle to a maximum of 12 cycles [92]. The extended carfilzomib infusion to weekly dosing was well tolerated. Besides being advantageous for patient convenience, the acceptable toxicity and PK allows for easier integration into subsequent combination therapy clinical trials [90,94].

The medications cyclophosphamide and etoposide are also standard drugs often used together for the treatment of cancer in children with solid tumors [95]. This prompted the development of an additional phase I trial that is currently evaluating carfilzomib with these two reagents for pediatric patients with relapsed/refractory solid tumors (NCT 02512926) [95].

### 5.3. Second-Generation PI: Ixazomib

Ixazomib was the first oral proteasome inhibitor and was approved by the FDA in 2015 as a second-generation PI in combination with lenalidomide plus dexamethasone for patients with MM who received as least one prior therapy (Table 1) [96]. Approval was based on a randomized, double-blind, placebo-controlled phase III trial (TOURMALINE-MM_1_) [107]. Patients with relapsed and/or refractory MM that received one to three prior therapies, were given either dexamethasone plus ixasomib (40 mg dexamethasone capsules once, orally, on days 1, 8, 15, and 21, plus 4 mg ixasomib capsules once, orally, on days 1, 8, and 15) or a placebo (placebo dexamethasone) over a 28-day cycle [107]. Ixazomib–dexamethasone significantly improved progression-free survival (PFS) compared with placebo dexamethasone (median 20.6 vs. 14.7 months, hazard ratio [HR] 0.74, *p*  =  0.01) [107].

#### 5.3.1. In Vitro

The in vitro sensitivity of canine and human OS cells to bortezomib and ixazomib was recently evaluated and OS cells from both species were found to be more sensitive to bortezomib than to ixazomib [45]. To further explore the inhibitory effect of ixazomib, Wilson-Robles et al. recently exposed two canine (MCKOS, Abrams) and two human (HOS, 143B) OS cell lines to this PI in vitro [97]. Ixazomib at a concentration of 10 μM was tested alone and in tandem with the STAT3 inhibitor SH4-54, based on the observation that downstream targets of STAT3 signaling were overexpressed in OS in both species [97]. All four cell lines were sensitive to ixazomib, while one human cell line (143B) and both canine cell lines were resistant to SH4-54 [97]. In terms of pro-tumorigenic traits, ixazomib was also better at inhibiting invasion compared to SH4-54 [97]. When tested together, co-treatment of ixazomib and SH4-54 demonstrated moderate inhibition against canine and human cell lines [97]. Similarly, Harris et al. found that ixazomib, added at concentrations between 0.1% and 10× the peak plasma concentration (C_max_ = 300 nM) of ixazomib, was toxic to canine (KRIB) and human (143B, KHOS) OS cells in vitro [98].

#### 5.3.2. In Vivo

Wilson-Robles et al. then tested ixazomib on canine MCKOS and human 143B cells in a murine xenograft model. Cells (1 × 10^6^ per mL) were injected subcutaneously into both right and left flanks of each mouse. Ixazomib, administered intraperitoneally at a dose of 10.7 mg/kg for four consecutive days followed by three days of rest, demonstrated inhibitory effects on growth and lung metastases [97]. Harris et al. tested ixazomib and bortezomib against xenografts of luciferase-expressing cell lines KRIB (KRIB-luc) and 143B (143B-luc) in athymic BalB/c nude mice [98]. Ixazomib, given at 5 mg/kg twice weekly for four weeks, but not bortezomib (1 mg/kg), was shown to slow metastases from KRIB-luc primary tumors and inhibit the growth of 143B-luc pulmonary and abdominal OS metastases. Ixazomib reportedly has enhanced solid tumor penetration compared to bortezomib [98], possibly as a result of its distinct physicochemical properties. For instance, ixazomib has a shortened proteasome dissociation t_1/2_, which is believed to play a critical role in the ability of this molecule to distribute well into tissues. Moreover, improved PK and PD tolerability allows this molecule to be administered at higher doses, resulting in greater blood and plasma concentrations and consequent tumor tissue exposures [108]. These data suggest that ixazomib may exert better single-agent activity against OS metastases than bortezomib and has the potential to improve outcomes for patients with metastatic OS [98].

#### 5.3.3. Clinical

It is well understood that food can change a drug’s bioavailability. This phenomenon prompted efficacy trials that evaluated the oral administration of ixazomib on an empty stomach [109]. After ixazomib was approved, Gupta et al. sought to conduct a phase I PK study to assess whether the PK of oral ixazomib would be altered if administered after a high-calorie, high-fat meal [109]. The results in patients with advanced solid tumors or lymphoma supports ixazomib being given on an empty stomach, at least 1 h before or at least 2 h after food [107]. Additionally, ixazomib has undergone further PK assessments in several trials (NCT 01830816, NCT 01953783), with a daily dose ranging between 3.0 and 4.1 mg [110,111].

Other ongoing trials are testing ixazomib as a co-treatment therapy. A phase I trial aims to find the highest tolerable dose of the combination of ixazomib and erlotinib that can be given to patients with advanced solid tumors (NCT 02942095) [99]. Erlotinib, a selective epidermal growth factor receptor tyrosine kinase inhibitor, is FDA-approved for the treatment of unselected recurrent non-small-cell lung cancer, though its use in advanced solid cancers is tentative [99]. Furthermore, vorinostat, a HDAC inhibitor, was evaluated in combination with ixazomib for patients with advanced p53 mutant malignancies (NCT 02042989) [100,101]. This trial was undertaken because prior preclinical studies showed that proteasome inhibition caused apoptosis both dependent and independent of the presence of wild-type p53. In addition, independent studies showed that HDAC inhibitors preferentially kill cells that harbor mutant p53, and that combined proteasome and HDAC inhibition synergize against cancer cells. The latter was linked to their capacity to modulate epigenetic gene expression, post-translational modifications, and protein degradation in the proteasome pathway, thus enhancing cellular stress and cell death [101]. Unfortunately, these results did not translate clinically, as none of the 59 patients harboring advanced, mutant p53-positive tumors of different origin had an objective response to this combination treatment. These include four patients with sarcomas.

### 5.4. Second-Generation PIs in Clinical Development: Oprozomib and Delanzomib

Newer PIs, oprozomib and delanzomib, were developed in efforts to improve the pharmacology and clinical efficacy, and reduce the toxicity seen in previous early generation PIs (Table 2) [112]. These PIs display enhanced binding affinity for proteasomal subunits, favorable pharmaceutical properties (i.e., oral bioavailability), and fewer adverse events [112]. Oprozomib is an orally bioavailable peptide epoxyketone-based, irreversible PI [112]. Delanzomib is a reversibly binding boronate-based PI with both oral and intravenous bioavailability [87]. Both PIs primarily bind to the β5 (CT-L) subunit of the proteasome and are being investigated in phase I/II trials [112].

#### 5.4.1. In Vitro

Oprozomib and delanzomib have been studied in canine (D17, OSCA8, OSCA40, OSCA78) and human (SaOS2, SJSA1, OS9, OS17) OS cells, which showed consistent sensitivity to bortezomib [45]. Carfilzomib and bortezomib demonstrate a slightly higher toxicity profile than the new PIs, with average IC_50_ values of 4.5 and 5.6 nM, respectively. In comparison, the average IC_50_ values of ixazomib, delanzomib, and oprozomib, were between 9.2 and 15.7 nM [45]. Canine PK and toxicity profiles of these drugs have yet to be published, but their peak plasma concentrations in humans were reported to be 1.9 to 5.1 μM for carfilzomib, 1.4 μM for oprozomib, and 800 nM for delanzomib [45]. These data show that concentrations of each drug that were highly toxic to the OS cells in vitro may be achievable in vivo, but this needs to be confirmed in mouse, canine, and human studies [45].

#### 5.4.2. Clinical

Currently, a phase I, open-label, dose escalation study is assessing the oral administration of oprozomib in patients with advanced refractory or recurrent solid tumors (NCT 01129349) [113]. An additional phase I, open-label, multicenter, dose-escalating study is assessing the safety, tolerability, PK, and pharmacodynamics (PD) of delanzomib given intravenously as a single agent in patients with advanced, incurable solid tumors (NCT 00572637) [114]. Both studies aim to identify the recommended dose for each PI to be used in phase II trials [113,114]. To the best of our knowledge, results from these trials have not been published.

### 5.5. Third-Generation PIs in Clinical Development: MG132

The latest work has investigated third-generation inhibitors for the treatment of OS. MG132 is a peptide aldehyde and a potent, reversible, cell-permeable 20S PI that is derived from a Chinese medicinal plant (Table 2) [115]. It inhibits the β5 (CT-L) activity of the proteasome [49].

#### 5.5.1. In Vitro

Most recently, a study examined the anticancer effects of MG132 against the human OS cell line U2OS [115]. The results show that MG132 suppressed proliferation and induced DNA damage, which led to increased apoptosis [115]. Interestingly, these events were accompanied by the downregulation of the NF-κB pathway, as well as cell cycle modulators and antiapoptotic proteins, including CDK2, CDK4, Bcl-xL, and Bcl-2 [115]. Moreover, MG132 treatment also resulted in the upregulation of proapoptotic proteins, including p21, p27, p53, and cleaved forms of caspase-3, -7, and -9 [115]. Another report also observed apoptotic bodies in MG132 treatments, with proteasome inhibition primarily causing cell arrest at the G_2_-M-phase [116]. However, these authors found increased activation of caspase-8, but did not observe caspase-3 or caspase-9 activity [116].

Human OS often harbors mutant p53 and contains a nonfunctional form of the Rb gene, two tumor-suppressor genes fundamental in controlling cell proliferation [117]. Taken together, researchers evaluated the effect exerted by p53 and Rb expression on MG132-induced apoptosis [49]. It was found that introducing the Rb gene into OS cells, such as SaOS2, exerts a protective influence against apoptosis, while the p53 introduction potentiates the apoptotic effect induced by MG132 [49].

The mechanism by which MG132 induces cell death has also been explored. Li et al. examined the effect of MG132 on TRAIL-induced apoptosis of a human OS cell line [61]. The results indicate that combination of MG132 and TRAIL resulted in the upregulation of DR5 expression and suppressed the invasion ability of OS cells significantly [61].

Of note, MG132 has also been shown to activate autophagy in lung [118] and breast cancer cells [119]. In the lung cancer cells, autophagy was shown to enhance sensitivity to the anti-angiogenic drug bevacizumab by facilitating the clearance of the protein disulfide isomerase anterior gradient 2 (AGR2), a pro-angiogenic protein overexpressed by many cancer types [118]. In the breast cancer cells, autophagy was associated to ER stress and suppressed apoptosis, suggesting that the combination of PIs with inhibitors of ER stress or autophagy may potentiate their cell death-inducing effects [119]. Such a combination of PIs might be worth exploring in OS, where autophagy has a documented role as a protective mechanism against cell death-inducing stimuli, such as chemotherapy [120,121].

#### 5.5.2. In Vivo

Cisplatin has been shown to be an integral part of the chemotherapeutic regimen in OS treatment; however, its use is hindered by chemotherapeutic resistance [58]. As such, exploring cisplatin in combination therapy is needed to circumvent the limitations of this reagent alone. Sun et al. examined the effects of MG132 in co-treatment with cisplatin in human OS xenografts of MG63 and HOS cells in nude mice [58]. The combination therapy showed significant inhibitory effects against tumor growth and exerted greater antitumor efficacy compared to the single-agent treatments [58]. The synergistic interaction between MG132 and cisplatin raises the possibility of testing this co-treatment clinically in OS patients. To the best of our knowledge there were no MG132 clinical trials ongoing by the time this manuscript was written.

### 5.6. Emerging Inhibitors

Up until this point, all of the PIs discussed for treating OS are covalent inhibitors. However, it is noteworthy to mention that covalent inhibitors have unstable chemical groups with high reactivity [57], potentially limiting their suitability for OS treatment [59,122]. PI-1840, a novel non-covalent PI, was synthesized with the exact purpose of overcoming this problem [122]. Studies have revealed that PI-1840 inhibits the growth of several tumor cell types by acting in a non-covalent manner (Table 2) [59]. Recently, the effects of PI-1840 were evaluated in MG63 and U2OS [57]. PI-1840 inhibited proliferation and induced apoptosis of these OS cell lines, partly by attenuating the NF-κB pathway [57]. Moreover, a reduction in migration and invasion capabilities of OS cells was also observed [57]. This suggests that PI-1840 could be a potentially effective treatment for OS.

The acquisition of drug resistance, a common outcome in many cancer therapies, is also a major hurdle in PI-based therapies [29,123,124]. Patients that initially respond to PIs targeting the 20S catalytic core of the proteasome almost always develop a resistance [29,123,124]. This has also prompted the search for novel PIs, which could potentially overcome this resistance [124]. Inhibitors directed towards the 19S regulatory subunit of the proteasome, especially the deubiquitinases (DUBs), are viable candidates in this regard [124]. This is due to their ability to bind to an alternative site on the proteasome [124]. DUBs in the human genome can be classified into subclasses based on their ubiquitin–protease domains, with ubiquitin-specific proteases (USPs) representing the largest class and major target in OS cells [125]. USPs, which play a vital role in the regulation of cellular responses to DNA damage, have been found to be overexpressed in OS tissue [125]. More recently, studies have targeted USPs, which have effectively inhibited the proliferation and invasion of human OS cells (Table 3) [126,127,128,129].

The scope of study related to DUBs in OS is fairly narrow, thus requires expansion. UCHL5 (or UCH37), USP14, and POH1 (Rpn11/PSMD14) are the three DUBs of the 19S proteasome that have been heavily investigated and targeted due to their promising properties on cancer cells [124]. As such, targeting these DUBs in OS cells should be a focus of future research. Of particular interest, b-AP15 was found to inhibit both UCHL5/UCH37 and USP14 [124]. Gene expression signatures of b-AP15-treated cells share similarities with bortezomib, but still target different proteasome subunits [124]. This trait allows b-AP15 to disrupt the cancer’s protective mechanism of forming aggresomes, a phenomenon observed when cells are exposed to bortezomib [124]. RA190 is another inhibitor targeting UCH37 [124]. RA170 has shown effectivity in MM cells resistant to bortezomib and in several preclinical cancer models, including MM, ovarian, cervical, and gastric cancers either alone or in combination with chemotherapy [124]. Besides inhibitors targeting UCH37 and USP14, other studies have explored Rpn11 inhibitor, capzimin, which has expressed activity in several cancer cell lines, including bortezomib-resistant cells [124]. Our group recently identified POH1/Rpn11/PSMD14 in extracellular vesicles released by canine OS explants and demonstrated pro-apoptotic, growth-inhibitory, and anti-migratory properties of capzimin in D17 and OVC-cOSA-31 cells, both derived from metastatic canine OS nodules [130]. Beyond these three DUBs, studies should also explore other potential DUBs, as well as corresponding target inhibitors, to optimize the potential for improving OS therapies.

## 6. Caveats and Unanswered Questions for Future Research

Although proteasome inhibition presents a novel and interesting therapeutic avenue in OS, there are several research questions and caveats that need to be explored in future studies.

### 6.1. Will the Clinical Success Experienced in Multiple Myeloma Be Achievable in OS Patients?

Although PI has been demonstrated to be clinically successful in MM, the extension of PI use to other cancers, particular solid tumors, has been questioned [131]. For instance, if cancer cells are highly dependent on protein quality-control mechanisms to sustain their highly proliferative nature and elevated rate of protein synthesis, then, in theory, almost all cancer cells should benefit from such therapies. However, this is likely not the case and MM cells’ heightened sensitivity to PI may be attributed to certain cell characteristics. First, MM cells are specialized to produce and secrete various immunoglobulins and cytokines [132]. As such, they have a well-established ERAD system and heavily rely on the proteasome to degrade misfolded proteins. PI can greatly skew the balance to proteotoxic stress and cause a terminal UPR. Second, MM cells have heightened levels and activity of NF-κB [133,134], in part due to mutations in genes that activate its signaling. Together, these two characteristics make MM a particularly sensitive target of proteasome inhibition. Another reason for a variable response to PI is due to differences in proteasome catalytic activity and subcellular localization across cancer types. A study that profiled the structure and function of proteasomes in breast, colorectal, and pancreatic cancer found that there were differences in proteasome subcellular distribution and catalytic activity. These differences may contribute to a heterogenous response to PIs [135].

It remains to be seen if this level of sensitivity will be demonstrated in OS. However, there are a few lines of evidence that could suggest that OS may be a good candidate for PI therapies. OS tumor biopsies and cell line studies indicate a high degree of genomic instability, with examples of chromosomal alterations and aneuploidy [136,137,138,139]. These genomic alterations can undoubtedly change the levels and folding properties of proteins being made, and as such, make them particularly reliant on protein quality-control mechanisms. In support of this hypothesis, a study conducted in yeast by Torres et al. found that aneuploid strains of yeast were sensitive to proteasome inhibition, while their wild-type counterparts were not [140]. Furthermore, aneuploid yeast are more susceptible to proteotoxic stress due to an increased number of protein aggregates [141]. Another reason OS may be a good candidate for PI therapies is due to mutations in phosphatase and the tensin homolog gene (PTEN). Loss of PTEN is common in both human and canine OS [80,142,143,144,145]. Jiang et al. found that PTEN status can influence sensitivity to bortezomib in cholangiocarcinoma [146]. PTEN-deficient cholangiocarcinoma cell lines tested in culture or grown in mice, as well as patients with this molecular tumor subtype, exhibited an increased response to bortezomib treatment [146]. This heightened response is believed to result from PTEN-deficient cells having a greater protein synthesis rate and reliance on protein-control mechanisms [146]. PTEN-deficient cells have lower levels of heat shock factor 1, a regulator of the heat shock protein response, a low capacity for protein folding in the ER, and increased protein aggresomes [146]. Additional research will have to include PTEN-deficient OS cell lines to confirm these findings. If validated, an additional step will be to test whether PTEN status could be used as a biomarker to identify candidates for PI-based therapies.

### 6.2. Is ER Stress and the UPR Advantageous in OS Progression and a Targetable Vulnerablility?

As mentioned above, PIs can induce apoptosis through a variety of different mechanisms, one of which is by inducing ER stress leading to an adaptive UPR. It is important to note that UPR can be both pro-apoptotic and pro-tumorigenic (see reviews [147,148]). The pro-tumorigenic effects can be mainly attributed to the ER stress proteins and chaperones that are upregulated as part of the UPR. A study that conducted proteomic profiling of OS tissue found that UPR proteins are elevated when compared to their respective non-malignant controls. These authors also noted, albeit only in a small number of patient samples, that higher levels of UPR proteins correlated with a more advanced disease stage and poor response to chemotherapy [149]. This correlation is likely attributed to these proteins playing key roles in metastatic progression and chemoresistance. In vitro studies found that OS cells upregulate the ER stress chaperone, glucose-regulated protein 78 (GRP78), to ensure cell survival and successful lung colonization, while its depletion decreases the metastatic burden [150]. Both GRP78 and ATF6α have been demonstrated to help cells survive chemotherapy treatment, through activating NF-κB signaling and downstream survival mechanisms [151,152]. GRP78 levels may also impact the ability of OS cells to respond to PIs, as knockdown of GRP78 greatly increased cells’ sensitivity to bortezomib [153]. These findings demonstrate that these molecules play a key role in mediating the response to therapy, and, thus, OS cell survival. This leads one to ask: could proteasome inhibition increase ER stress and UPR proteins and thus promote OS survival? Or would proteasome inhibition add to the stress that OS cells are already experiencing, reaching a proteotoxic threshold that can ultimately trigger apoptosis? Further studies will need to elucidate this relationship and whether PI exacerbates ER stress and UPR in a pro-survival or cytotoxic manner. As proteasome inhibitors impact several signaling pathways and cellular molecules, it is possible that cell death can also be triggered by alternative mechanisms, especially when combined with other therapies. One possibility of such a combination treatment is through simultaneously inhibiting heat shock proteins (HSPs) and the proteasome. HSPs are molecular chaperones which aid in protein folding and mitigating protein damage as a result of stress. Inhibition of both HSPs and the proteasome may enhance the anti-tumor potential of both compounds by causing a toxic level of protein accumulation, leading to apoptosis [154].

In OS research, most studies rely on oversimplified two-dimensional (2D) assessments that do not fully recapitulate the changes in signaling that promote OS progression. These models fail to acknowledge the dynamic environment in which cancer cells grow, including proper cell–cell and cell–extracellular matrix interactions that influence cancer progression [155]. Therefore, incorporating three-dimensional (3D) culture models that mimic better the physical microenvironment of OS cells, in the design of experiments for PI testing, may provide a more realistic picture of their anti-tumor effects.

### 6.3. Do Proteasome Inhibitors Have Immunomodulatory Properties and Are These Contributing to Their Mechanism of Action?

The aforementioned in vivo studies with PIs were performed in xenograft models, making it difficult to extrapolate these findings to individuals with a functional immune system. A recent study by Benvenuto et al. found that the number of B lymphocytes, CD4^+^ and CD8^+^ T cells, macrophages, and natural killer cells increased within the tumor microenvironment upon bortezomib treatment in a head and neck cancer mouse model [156]. It is important to know if PIs can modulate immune cell populations in the OS tumor microenvironment, especially within metastatic lesions, and whether this could be exploited to design more effective treatments for metastatic disease.

## 7. Limitations

Although there has been clinical success with proteasome-targeted therapies for cancer treatment, data indicate limitations associated with their use in solid tumors. Like other therapeutic options, a subset of patients may respond to PIs, while others do not. Even those who express an initial response inevitably develop resistance over time [87]. Initial studies have established genetic mutations in PSMB5 (encoding proteasome subunit β5) as the underlying cause of PI resistance in vitro [157]. However, increasing evidence emphasizes the contribution of non-mutational epigenetic mechanisms. Ge et al. recently found that drug-resistant cells can not only emerge from the treatment-mediated selection of subpopulations that present at the start of therapy, but also from epigenetic alterations under therapy stress [157]. These authors further suggested combination therapy with HDAC inhibitors and/or high-dose intermittent therapy [157].

The lack of therapeutic efficacy of PIs against solid cancers, such as OS, has often been attributed in part to their poor PK profiles, including their short circulation time and insufficient distribution to proteasome targets within solid tumor tissues [87]. Bortezomib, for example, has shown clinical efficacy in MM and ML, but has yet to exhibit strong activity in solid tumors [87]. This is perhaps due to its inability to penetrate into tissues and achieve therapeutically relevant concentrations at the β subunit target sites. Focusing on the structural scaffolds of PIs may be required to address these limitations and expand the utility of existing PIs [87].

It is critical to examine both the PK and PD profiles of PI candidates to successfully bridge the gap between initial preclinical results and eventual clinical outcomes. PIs have been associated with adverse effects from peripheral neuropathy to cardiovascular complications, such as hypertension and heart failure [70]. Second-generation PIs have demonstrated a reduction in the incidence of peripheral neuropathy, the major dose-limiting toxicity of bortezomib. Oral PIs, such as ixazomib, are now available, providing more convenient administration and better tolerability [108]. However, even these PIs have gastrointestinal side effects [109]. Moving forward, efforts should be made to further our understanding of underlying mechanisms, while also identifying potential biomarkers to predicate efficacy or toxicity [70].

## 8. Conclusions

The heterogeneity of OS is widely acknowledged in both clinical and molecular reports [3,6,14]. Analyses detailing disease properties have highlighted poor patient prognoses amongst OS metastatic or metastasis-prone populations. Considering the limited therapeutic progress made in treating OS, investigating alternative treatment plans is critical for improving patient outcomes.

The use of PIs in cancer has cultivated interest amongst many researchers due to its promising potential as an anti-cancer treatment. Malignant cells, which are characterized by rapid proliferation and enhanced survival, are assumed to require more proteasomes than normal cells for protein homeostasis and to sustain their efficient biological activities [24]. Specifically, OS tumors have been found to possess multiple genetic and cell-signaling aberrations, which may make PIs that affect multiple cellular pathways more effective than agents that only target one signaling pathway. Bortezomib, carfilzomib, and ixazomib have shown high effectivity in OS cells [45]. Of the anti-OS drugs tested, bortezomib appears to be favored, as it significantly inhibits cell proliferation and induces tumor regression [45,72,73]. However, work has indicated that ixazomib may be a better single agent against OS metastases [98]. These findings support further investigation of PI-based therapies in OS.

With limited human tissue available for study, mouse models provide a valuable tool to investigate the underlying mechanisms of tumor initiation, progression, metastatic events, and test therapeutic interventions. While no such models have yet to fully recapitulate all aspects of OS, there is no doubt they have provided valuable insights on the biology of OS [45,75,77]. However, spontaneous and secondary OS is common in large canines, more so than in humans, making dogs an attractive model to improve our understanding of disease vulnerabilities in both species [45,75,77]. As new therapies continue to be evaluated, it is important to consider the value of canine OS trials and how they can improve the likelihood of new treatments being successful in human OS patients.

Research has consistently shown the effectiveness of PIs as an anti-cancer agent. Despite their sometimes impressive clinical activity, development of treatment resistance is common [124]. Importantly, response to bortezomib, the leading PI agent for cancer treatment, is highly dependent on tumor vascularity [123]. In efforts to counteract this problematic phenomenon, Zuccari et al. have explored strategies to overcome bortezomib resistance and improve the PI’s bioavailability by packing the drug into liposomes that permeate preferentially through tumor vasculature [123]. This strategy was found to be far more effective at treating xenograft solid tumors than unpackaged bortezomib [123]. As such, additional research on the use of liposomes or alternative nanocarriers to improve treatment outcomes will be beneficial. Furthermore, researchers have started to investigate strategies outside proteasome 20S inhibitors to overcome acquired drug resistance. As noted, inhibitors of 19S proteasome regulatory particles, especially DUBs, are promising agents [124] and deserve further testing, both as single agents and in combination with proteasome 20S inhibitors.

Continuing to analyze the PIs discussed, as well as novel PIs against OS cells, is necessary to identify the optimal treatment strategy for OS patients. From a neoadjuvant perspective, PIs may represent opportunities to enhance patient responses when combined with chemotherapeutic agents. Nonetheless, the ultimate goal is to improve patient care, treatment, and quality of life, while prolonging life for patients with OS, ideally by preventing and/or effectively targeting metastatic disease.

## Figures and Tables

**Table 1:** Key findings from preclinical and clinical research that has explored FDA-approved PIs in both human and canine OS.

Agent	Disease Agent is Approved for	Key Findings from In Vitro Studies	Key Findings from In Vivo Studies	Key Findings from Human Trials or Current Clinical Trials
Bortezomib [MLN-341; PS-341; Velcade^®^]	First-line therapy for MM in combination with an alkylating agent and a corticosteroid. Second-line therapy for MM alone. Second-line therapy for MCL alone, in patients who received at least one previous therapy [81].	OS cell line sensitivity: Canine (D17, OSCA8, OSCA40, OSCA78) and human (SaOS2, SJSA1, OS9, OS17) cell lines treated for 48 hrs showed high sensitivity [45].	Human OS xenograft apoptosis: 143B luciferase-expressing cells grown in Nu/Nu mice. After 3 weeks, bortezomib treatment reduced growth and induced OS cell apoptosis. These results correlated with increased immunoreactivity for BAX [82].	Human Trials: A multicenter phase II study of bortezomib in recurrent or metastatic sarcoma patients. All patients had not received chemotherapy for metastatic disease. One leiomyosarcoma patient had a partial response. A single OS patient was included but their response was not specified [83].
		Canine OS apoptosis and cell cycle arrest: Bortezomib inhibited proteasome activity and caused caspase-dependent cell death after treatment for 24 hrs. G2 cell cycle arrest occurred after 7 to 24 h [45].	Co-treatment in Human OS xenografts: KHOS/NP cells were injected into Nu/Nu mice. The combination of bortezomib and doxorubicin resulted in significant tumor growth inhibition and activated the ROS and *p*-eIF2α/ATF4/CHOP axis in the UPR pathway [84].	Ongoing clinical trials: Bortezomib is being investigated alone (Phase II; NCT00027716) and in combination with the chemotherapeutic agent gemcitabine hydrocholoride (Phase II; NCT00620295) in patients with advanced or metastatic tumors [85,86].
		Human OS apoptosis and autophagy: Bortezomib treatment of HOS cells for up to 48 h induced growth inhibition in a time- and dose-dependent manner, and autophagy and apoptosis in a dose-dependent manner [47].		
		Co-treatment in canine OS: Bortezomib in combination with doxorubicin or carboplatin exerts more potent cytotoxicity than either agent alone on canine OS cells [45].		
Carfilzomib [PR-171; Kyprolis^®^]	Approved as a second-line therapy for relapsed and/or refractory MM [87].	OS cell line sensitivity: Canine (D17, OSCA8, OSCA40. OSCA78) and human (SaOS2, SJSA1, OS9, OS17) cell lines exposed to carfilzomib for 48 hrs respond comparably to bortezomib [45]. An average of >95% cytotoxic effect by carfilzomib in both canine (Abrams, Moresco, D17, D418) and human (143B, MG63, SAOS, U2OS, 17-3X) OS cells [88].	Co-treatment in OS xenografts: K7M2 or SAOS2-LM7 luciferase-expressing cells were injected into BalB/c or NSG mice, respectively. Carfilzomib, as a single agent, had no effect on primary or metastatic OS growth. However, the combination of carfilzomib and panobinostat attenuated metastatic growth [89].	Human Trials: Patients with normal hepatic function (normal) or hepatic impairment (mild, moderate, or severe) received carfilzomib infusions in 28-day cycles. Exacerbation of hepatic disfunction was observed in patients with mild and moderate hepatic impairment versus normal hepatic function patients. However, differences were not statistically significant [90].
		Effectivity in cells with treatment resistance and metastatic properties. Carfilzomib had cytotoxic effects on pediatric solid tumor cell lines, including OS cells. Combination with chemotherapeutic agents enhanced the effects [91].		Ongoing clinical trials evaluating safety, tolerability, and PK: A phase I study (NCT01949545) aims to find the safest dose level of carfilzomib in advanced solid tumors when given over a different period of time (days 1, 8, 15 of a 21-day cycle) compared to the typical dosing schedule (dosed on days 1, 2, 8, 9, 15, and 16 of a 28-day cycle to a maximum of 12 cycles) [92]. A phase 1b/2 study (NCT00531284) is evaluating the overall response rate (ORR) after four cycles of carfilzomib in patients with relapsed solid tumors, MM, or lymphoma [93].
		Co-treatment in human OS: Carfilzomib-induced cell death was enhanced when combined with MAPK inhibitors U0126, SP00125, or SB203580 in OS cells. Inhibition of ERK1/2 or JNK MAPK pathways significantly decreased the expression of anti-apoptotic Bcl-2 proteins [94].		Ongoing clinical trials examining co-treatments: In a phase I trial (NCT02257476), patients receive dexamethasone prior to weekly doses of carfilzomib over a 21-day cycle [92]. In another phase I trial (NCT02512926), pediatric patients with relapsed and/or refractory tumors receive carfilzomib in combination with cyclophosphamide and etoposide to examine dose-limiting toxicities (DLTs) until the maximum tolerated dose (MTD) is reached [95].
Ixazomib [MLN-9708; Ninlaro^®^]	Approved in combination with lenalidomide and dexamethasone for the treatment of MM after at least one prior therapy [96].	OS cell line sensitivity: Canine (D17, OSCA8, OSCA40, OSCA78) and human (SaOS2, SJSA1, OS9, OS17) cell lines were incubated for 48 h with ixazomib and cells showed less sensitivity in comparison to bortezomib [45].	Single agent in OS xenografts: Canine (MCKOS and Abrams) and human (HOS and 143B) cells were injected into athymic nude female mice. Ixazomib inhibited growth and metastases in 143B cells [97].	Human Trials: A phase I trial assessed whether the PK of ixazomib would be altered if administered after a high-calorie, high-fat meal. The results support the administration of ixazomib on an empty stomach, at least 1 h before or at least 2 h after food [95].
		Co-treatment in OS cells: Ixazomib alone and in combination with SH4-54 [97].	Co-treatment in OS xenografts: The combination of ixazomib with SH4-54 inhibited growth of canine MCKOS cells grown bilaterally in the flank of athymic nude mice [97]. In xenografts of luciferase-expressing KRIB or 143B OS cells in athymic nude mice, neither ixazomib nor bortezomib reduced primary KRIB tumor growth, but both inhibited pulmonary metastatic growth. Only ixazomib slowed KRIB metastases and inhibited the growth of 143B pulmonary and abdominal metastases, significantly enhancing the survival of mice injected with 143B cells [98].	Ongoing and completed clinical trials: A phase I trial (NCT02942095) is assessing the MTD of ixazomib in combination with erlotinib in patients with advanced cancer over a 28-day cycle [99]. In a completed phase I trial (NCT02042989), patients with advanced p53 mutant malignancies were administered ixazomib in combination with vorinostat over a 28-day cycle [100]. This did not elicit an objective response in any of the patients and was associated with poor PFS and overall survival [101].

**Table 2:** Key findings from preclinical and clinical research on canine and human OS, with PIs that are in clinical development.

Agent	Key Findings from In Vitro Studies	Key Findings from In Vivo Studies	Key Findings from Human Trials or Current Clinical Trials
Oprozomib [ONX-0912] and Delanzomib [CEP-18770]	OS cell line sensitivity: IC_50_ in canine (D17, OSCA8, OSCA40, OSCA78) and human (SaOS2, SJSA1, OS9, OS17) cell lines were <10 nM for both inhibitors, but 2–3 times higher than the IC_50_ for bortezomib [45].	No data on the efficacy of oprozomib and delanzomib in vivo to date.	Ongoing clinical trials: A phase I study (NCT01129349) is assessing the oral administration of oprozomib in patients with advanced refractory or recurrent solid tumors [113]. Another phase I trial (NCT00572637) is assessing the safety, tolerability, PK, and PD of delanzomib given intravenously as a single agent in patients with advanced, incurable solid tumors [114].
MG132	Human OS apoptosis: Suppressed proliferation and induced apoptosis in human (U2OS) OS cells. This is accompanied by the downregulation of the NF-κB pathway and anti-apoptotic proteins. Its effect on TRAIL-induced apoptosis in human (OS732) OS cells associates with upregulation of DR5 expression and suppression of invasion capabilities [115].	Co-treatment in OS cells: The combination of MG132 with cisplatin significantly inhibited tumor growth with greater efficacy than single-agent treatments in MG-63 and HOS xenografts in Balb/c nude mice [58].	No current data on the efficacy of MG132 in clinical trials.
	Influence of Rb and p53 on apoptosis: The rescue of Rb gene expression into human (SaOS2) OS cells protects against MG132-induced apoptosis, while re-expressing p53 potentiates the apoptotic effect induced by MG132 [49].		
PI-1840	Human OS apoptosis and autophagy: Inhibited the proliferation and induced apoptosis of MG-63 and U2-OS human OS cells, partly due to attenuation of the NF-κB pathway. Induced autophagy, and inhibiting autophagy led to enhanced survival of U2-OS cells. Hindered migration and invasion of the above OS cell lines [57].	No data on the efficacy of PI-1840 in vivo to date.	No reports on the efficacy of PI-1840 in clinical trials to date.

**Table 3:** Key findings from preclinical research that has explored USP inhibitors in both in vitro and in vivo studies with OS cells.

Targeted USP	USP Inhibitor	Key Findings from In Vitro Studies	Key Findings from In Vivo Studies
USP9x	Neogambogic acid (NGA)	NGA significantly inhibited the proliferation of OS cells and promoted ubiquitin-mediated proteasome degradation of SOX2. USP9x was identified as a deubiquitinase for SOX2, and NGA directly interacts with USP9x in cells. Knockdown of USP9x inhibited the proliferation and colony formation of OS cells [126].	Knockdown of USP9x inhibited the growth of OS xenografts in mice [126].
USP1	Lentiviral vector harboring RNA interference (RNAi) targeting USP1 [127].	Significant suppression of the mRNA and protein expression of USP1 in U2OS cells, resulting in inhibition of cell growth, colony formation, and invasion. The suppression of USP1 expression downregulated the expression of many proteins, including Bcl-2 [127].	No in vivo studies with this or similar viral vectors to date.
	MicroRNA (miR)-192-5p	Low miR-192-5p levels in OS tissues and cell lines (143B, U2OS, hFOB) associate with high levels of USP1. Upregulating miR-192-5p expression inhibited cell proliferation, apoptosis, migration, and invasion, and increased OS cell sensitivity to cisplatin. USP1 was observed to be a direct target gene of miR-192-5p in OS. Upregulating USP1 promoted cell proliferation, migration, and invasion, and decreased cell chemo-sensitivity. This was partially reversed via the overexpression of miR-192-5p in OS cell lines [128].	No in vivo testing conducted with miR-192-5p to date.
USP17	To our knowledge, USP17 does not have a defined inhibitor. USP17 expression has only been correlated with the stabilization of tumor-suppressor proteins.	USP17 was upregulated in OS tissues and cell lines (MG-63, U2OS). In the latter, it was found to promote proliferation, as well as migration and invasion, via SMAD4-mediated epithelial-mesenchymal transition [129].	No in vivo testing involving USP17 inhibition to date.
